# Efficacy and safety of SCT200 in patients with recurrent or metastatic head and neck squamous cell carcinoma after platinum failure: A phase 2 study^☆^

**DOI:** 10.1016/j.cpt.2026.01.001

**Published:** 2026-01-10

**Authors:** Yuankai Shi, Qingyuan Zhang, Wei Wang, Jianhua Shi, Xiaodong Zhu, Kunyu Yang, Lin Wang, Guoqing Li, Peiguo Wang, Lin Gui

**Affiliations:** a Department of Medical Oncology, Beijing Key Laboratory of Key Technologies for Early Clinical Trial Evaluation of Innovative Drugs for Major Diseases, National Cancer Center/National Clinical Research Center for Cancer/Cancer Hospital, Chinese Academy of Medical Sciences & Peking Union Medical College, Beijing 100021, China; b Ward One of Mammary Department, Harbin Medical University Cancer Hospital, Harbin, Heilongjiang 150081, China; c Department of Medical Oncology, Digestive and Urinary Unit, The Affiliated Cancer Hospital of Xiangya School of Medicine, Central South University/Hunan Cancer Hospital, Changsha, Hunan 410013, China; d Department of Internal Medicine, Linyi Cancer Hospital, Linyi, Shandong 276000, China; e Department of Radiation Oncology, Guangxi Medical University Cancer Hospital, Guangxi Zhuang Autonomous Region, Nanning, Guangxi 530021, China; f Department of Cancer Center, Union Hospital, Tongji Medical College, Huazhong University of Science and Technology, Wuhan, Hubei 430022, China; g Department of Oncology, Hainan General Hospital, Haikou, Hainan 570311, China; h Department of Radiation Oncology, Jiangxi Cancer Hospital, Nanchang, Jiangxi 330029, China; i Department of Radiation Oncology, Tianjin Medical University Cancer Institute & Hospital, Tianjin 300060, China

**Keywords:** Head and neck squamous cell carcinoma, Epidermal growth factor receptor, Monoclonal antibodies, Recurrent or metastatic

## Abstract

**Background:**

There are limited treatment options for patients with recurrent or metastatic head and neck squamous cell carcinoma (R/M HNSCC) progressing after platinum-based therapy. Epidermal growth factor receptor (EGFR)-targeting monoclonal antibodies (mAbs) have demonstrated efficacy in R/M HNSCC. This phase 2 study evaluated the efficacy and safety of SCT200, a novel fully humanized EGFR mAb, in Chinese patients with R/M HNSCC progressing after platinum-based therapy.

**Methods:**

This was a single-arm, multi-center phase 2 study, which enrolled patients with R/M HNSCC who had progressed after ≥2 cycles of platinum-based therapy. All patients received SCT200 at 6.0 mg/kg weekly for the first 6 weeks, followed by 8.0 mg/kg every 2 weeks until disease progression or unacceptable toxicity. The primary endpoint was the objective response rate (ORR).

**Results:**

Between November 14, 2018, and April 2, 2020, 36 patients were enrolled. The ORR was 13.9% (5/36, 95% confidence interval [CI]: 4.7–29.5), the complete response rate was 2.8% (1/36), while the partial response rate was 11.1% (4/36). The disease control rate was 69.4% (25/36, 95% CI: 51.9–83.7). The median time to response was 1.38 months (95% CI: 0.99–2.60). The median duration of response was 5.95 months (95% CI: 2.60–not reached [NR]). The median progression-free survival and overall survival were 3.98 months (95% CI: 2.76–5.32) and 8.77 months (95% CI: 6.64–NR), respectively. Grade ≥3 treatment-related adverse events occurred in 44.4% (16/36) of patients, with the most common being hypomagnesemia (7/36, 19.4%, 95% CI: 8.2–36.0), hypokalemia (3/36, 8.3%, 95% CI: 1.8–22.5), rash (2/36, 5.6%, 95% CI: 0.7–18.7), and anemia (2/36, 5.6%, 95% CI: 0.7–18.7).

**Conclusions:**

This study demonstrated promising efficacy and a manageable safety profile of SCT200 in patients with R/M HNSCC progressing after platinum-based chemotherapy.

**Trial registration:**

Clinical Trials.gov, NCT03713372; https://www.clinicaltrials.gov.

## Highlights

• SCT200 is a novel, fully humanized anti-epidermal growth factor receptor monoclonal antibody.
• The objective response rate, disease control rate, median progression-free survival, and median overall survival of SCT200 was 13.9% (5/36, 95% CI: 4.7–29.5), 69.4% (25/36, 95% CI: 51.9–83.7), 3.98 months (95% CI: 2.76–5.32), and 8.77 months (95% CI: 6.64–not reached), respectively, for patients with recurrent or metastatic head and neck squamous cell carcinoma progressing after platinum-based therapy.
• SCT200 had a manageable safety profile, with primarily skin and electrolyte adverse events.

## Graphical abstract

**Figure:**
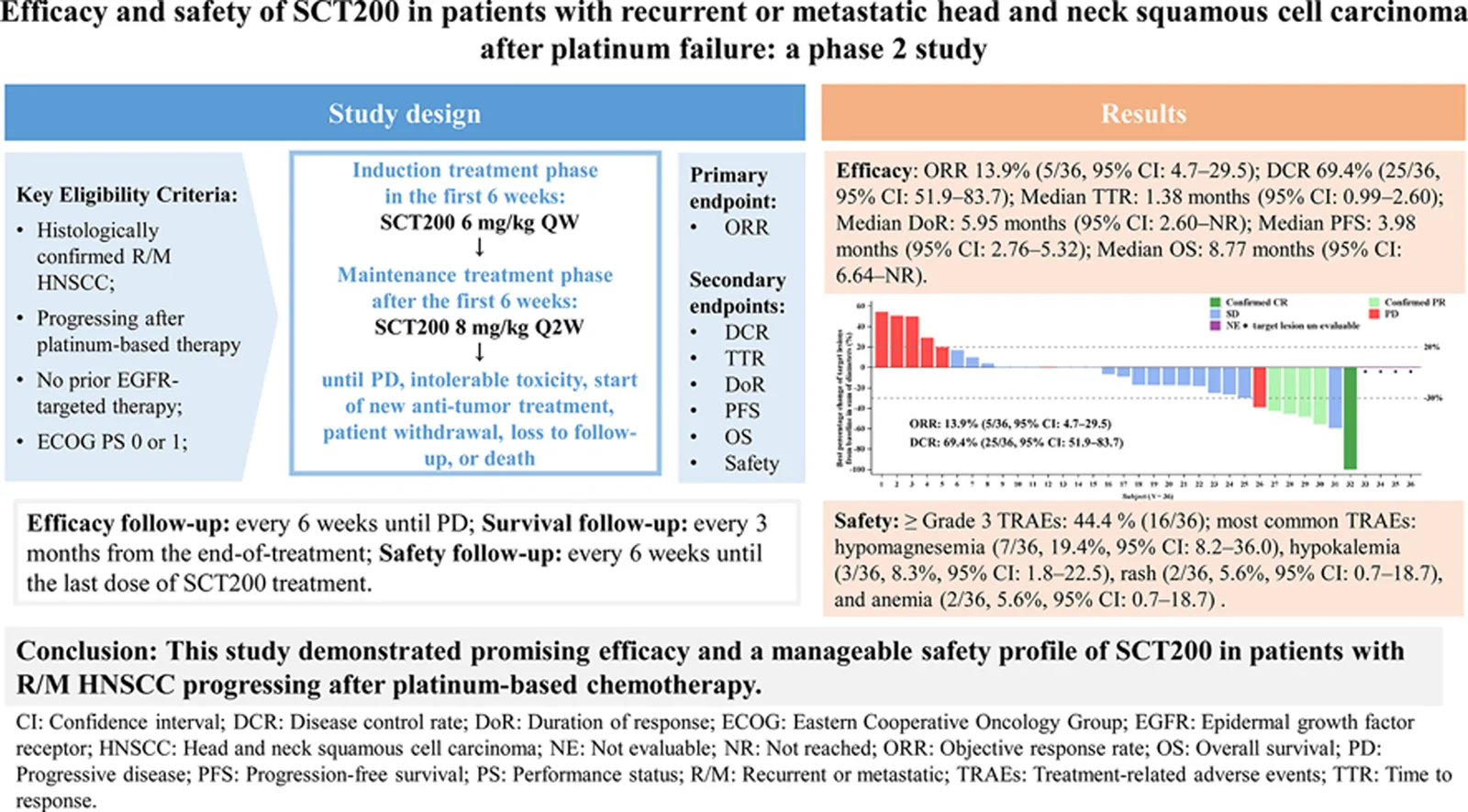

## Introduction

Head and neck tumors include cancers of the lip, oral cavity, oropharynx, nasopharynx, hypopharynx, and larynx. In 2022, there were an estimated 891,453 new cases and 458,107 deaths globally, with 139,179 new cases and 75,642 deaths in China.^1^^,^^2^ Approximately 90% of head and neck tumors are squamous cell carcinoma.^3^ More than 65% of patients with head and neck squamous-cell carcinoma (HNSCC) ultimately develop recurrence or distant metastasis.^4^ First-line systemic treatment for this patient population primarily consists of platinum-based chemotherapy combined with either epidermal growth factor receptor (EGFR) monoclonal antibodies (mAbs) or programmed cell death protein 1 (PD-1) inhibitors.^5^, ^6^, ^7^, ^8^, ^9^ For second-line systemic treatment, options include EGFR mAbs, PD-1 inhibitors, or single-agent chemotherapy such as methotrexate if these agents were not utilized in the first-line setting.^10^

EGFR plays a pivotal role in regulating cell proliferation, differentiation, division, survival, and cancer progression through its signaling cascade.^11^ *EGFR* overexpression or mutations occur in 80–90% of HNSCC and correlate with poor prognosis.^12^, ^13^, ^14^ Cetuximab is a chimeric human-murine anti-EGFR immunoglobulin G (IgG)-1 mAb that was approved by the U.S. Food and Drug Administration (FDA) on March 1, 2006, for the second-line treatment of patients with HNSCC following platinum-based therapy. However, there are currently no EGFR mAbs approved for this indication in China.

SCT200 is a fully humanized anti-EGFR mAb developed by Sinocelltech Ltd. (Beijing, China). It binds to tumor cells expressing EGFR and exerts its anti-tumor effects through complement-dependent cytotoxicity (CDC) and antibody-dependent cell-mediated cytotoxicity (ADCC). *In vitro*, SCT200 demonstrated anti-proliferative effects. In phase 1 and phase 2 studies, SCT200 has demonstrated preliminary anti-tumor efficacy and a manageable safety profile in patients with advanced esophageal squamous cell carcinoma and metastatic colorectal cancer.^15^, ^16^, ^17^

Currently, no clinical studies in China have evaluated an EGFR mAb as a second-line treatment for HNSCC. This study aimed to evaluate the efficacy and safety of SCT200 monotherapy in patients with recurrent or metastatic (R/M) HNSCC progressing after platinum-based therapy.

## Methods

### Study design

This was a multicenter, single-arm phase 2 study that recruited patients from 12 hospitals in China. Eligible patients were aged 18–75 years, with histologically or cytologically confirmed HNSCC who had progressed after ≥2 cycles of platinum-based chemotherapy or were intolerant to its adverse events (AEs). Patients were required to have at least one measurable lesion according to the Response Evaluation Criteria in Solid Tumors (RECIST) version 1.1. Patients were required to have an Eastern Cooperative Oncology Group (ECOG) performance status (PS) score of 0 or 1 and an expected survival of at least 3 months. The main exclusion criteria were as follows: patients with central nervous system metastases or a history of central nervous system metastases; a history of other malignant tumors; prior treatment with an EGFR mAb or small molecule EGFR inhibitors; and having received anti-tumor therapy within 4 weeks prior to enrollment.

### Treatment

All enrolled patients received SCT200 at 6.0 mg/kg intravenously weekly for the first 6 weeks. After that, the dosage was adjusted to 8.0 mg/kg every 2 weeks and continued until progressive disease (PD), intolerable AEs, the start of a new anti-tumor treatment, patient withdrawal, loss to follow-up, or death. SCT200 treatment was also discontinued in cases of more than two consecutive dosing interruptions or if the investigator determined that continuation was no longer appropriate.

### Assessment

Efficacy assessments were conducted according to RECIST version 1.1 using enhanced computed tomography every 6 weeks until PD, the start of new anti-tumor treatment, patient withdrawal, loss to follow-up, or death. AEs were assessed according to the National Cancer Institute Common Terminology Criteria for Adverse Events version 5.0 throughout the entire treatment period. Body weight, vital signs, and physical examinations were assessed prior to every dose administration (weekly for the first 6 weeks, every 2 weeks thereafter), and the end-of-treatment (EOT) visit. Meanwhile, safety assessments, including ECOG PS score, electrocardiogram, and laboratory tests, were carried out before the first dose of SCT200 was administered, and conducted every 6 weeks thereafter, and at the EOT visit within 14 days after the last dose of SCT200 treatment. Blood samples for immunogenicity assessment were collected within 7 days before the first dose of SCT200, every 6 weeks thereafter (prior to dosing), and at the EOT visit within 14 days after the last dose of SCT200 treatment. Survival follow-up was conducted every 3 months after the safety follow-up/disease progression follow-up.

### Endpoints

The primary endpoint of this study was the objective response rate (ORR), defined as the proportion of patients with a confirmed complete response (CR) or partial response (PR). Secondary endpoints included the disease control rate (DCR), time to response (TTR), duration of response (DoR), progression-free survival (PFS), overall survival (OS), safety, and immunogenicity. DCR was defined as the proportion of patients with CR, PR, or stable disease (SD). TTR was defined as the duration from the date of administration of the first dose of SCT200 to the date of the first recorded CR or PR. DoR was defined as the duration from the date of the first recorded CR or PR to the date of the first recorded PD or death from any cause. PFS was defined as the duration from the date of the first dose of SCT200 to the date of the first recorded PD or death from any cause. OS was defined as the duration from the date of the first dose of SCT200 to the date of death.

### Statistical analysis

Historical data indicated that single-agent methotrexate yielded an ORR of approximately 4% (95% confidence interval [CI]: 2%–8%).^18^ This value was adopted as the reference null rate (*p*_*0*_ = 0.04). We aimed to detect a target ORR of 15% (*p*_*1*_ = 0.15) for SCT200 under an exploratory, single-arm design. Using a one-sample proportion test (normal approximation) with a one-sided *α* value of 0.05 and a power of 80%, the minimum required sample size was 32 patients. Accounting for an estimated dropout rate of 12%, a total of 36 participating patients were planned for enrollment.

The full analysis set (FAS) comprised all patients who received at least one dose of SCT200 treatment and was used for efficacy analyses. The safety set (SS) included all patients who received at least one dose of SCT200 treatment and underwent at least one post-treatment safety assessment. The SS was used for safety analyses. Statistical analyses were performed using SAS software version 9.4 (SAS Institute, Cary, NY, USA). TTR, DoR, PFS, and OS were analyzed using the Kaplan–Meier method, with median survival times and 95% CIs calculated using the Brookmeyer and Crowley method. The 95% CI for ORR was calculated using the Clopper-Pearson exact method for the binomial distribution.

## Results

### Patient baseline characteristics

**Figure 1:**
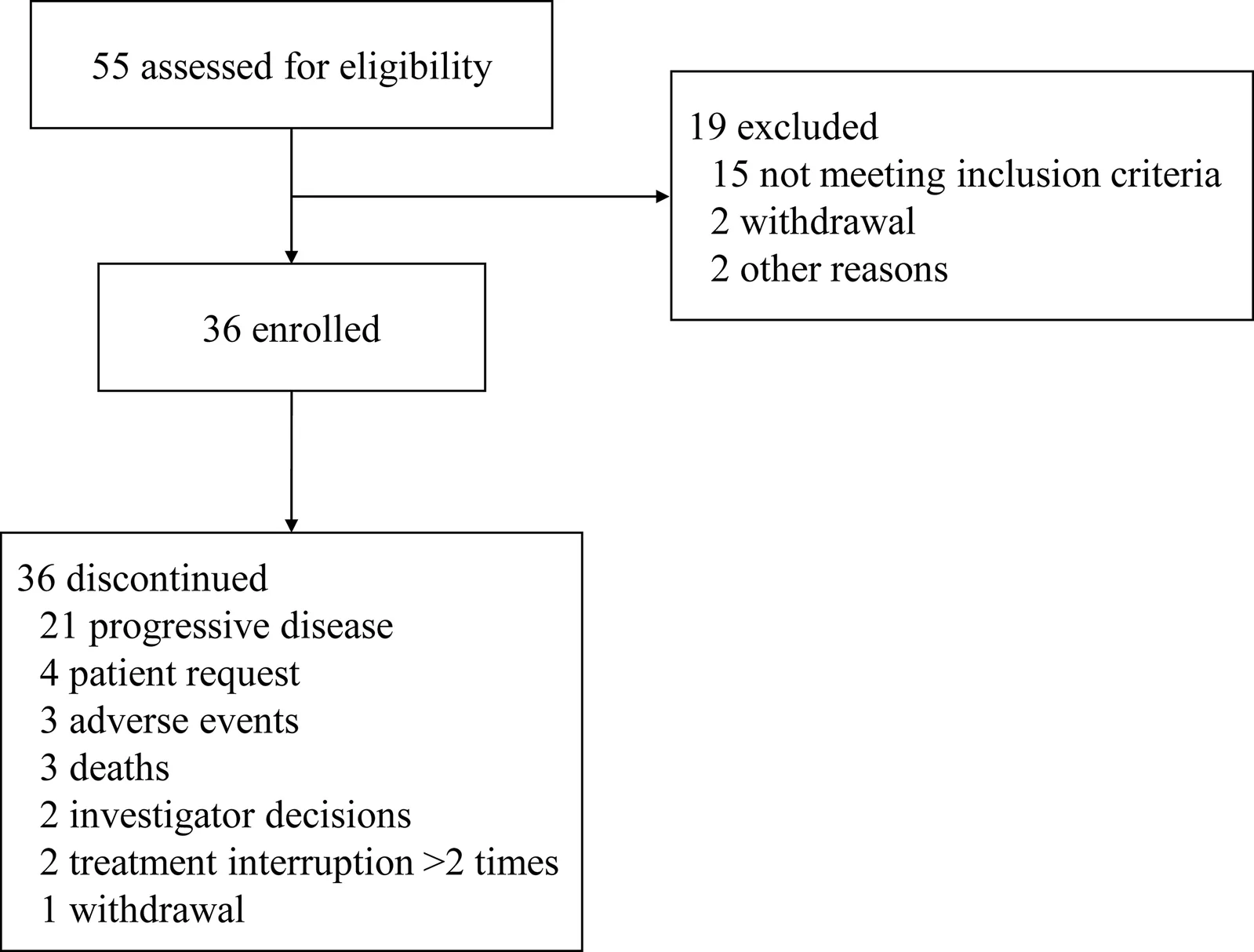
Trial flowchart of the phase 2 study evaluating SCT200 in patients with recurrent or metastatic head and neck squamous cell carcinoma progression after platinum-based therapy in the full analysis set.

From November 14, 2018, to June 28, 2019, a total of 55 patients were screened, and 36 were enrolled [Figure 1]. All patients received at least one dose of SCT200 and were included in the FAS. The median age was 60 years (range: 45–72), with 94.4% (34/36) being men, 38.9% (14/36) of patients having cancers of the larynx, 38.9% (14/36) of patients having metastatic disease, and 64.3% (9/14) of patients with cancers that metastasized to the lungs [Table 1]. All patients had previously received platinum-based therapy.

**Table 1:** Baseline demographic and clinical characteristics of patients with platinum-refractory recurrent or metastatic HNSCC treated with SCT200 in the phase 2 trial in the FAS (*N* = 36).

Characteristic	Value
Age, years	
Mean ± standard deviation	58.2 ± 7.4
Median (range)	60.0 (45, 72)
Sex, *n* (%)	
Male	34 (94.4)
Female	2 (5.6)
ECOG PS, *n* (%)	
0	6 (16.7)
1	30 (83.3)
Primary tumor site, *n* (%)	
Oral cavity	6 (16.7)
Oropharynx	5 (13.9)
Hypopharynx	8 (22.2)
Larynx	14 (38.9)
Other	3 (8.3)
Metastatic site, *n* (%)	
Brain	0 (0.0)
Lung	9 (64.3)
Liver	2 (14.3)
Bone	5 (35.7)
Other	4 (28.6)
Recurrent, *n* (%)	
Yes	21 (58.3)
No	15 (41.7)
Previous lines of chemotherapy	
Median (range)	1.0 (1, 5)
1, *n* (%)	26 (72.2)
2, *n* (%)	7 (19.4)
≥3, *n* (%)	3 (8.3)

ECOG: Eastern Cooperative Oncology Group; FAS: Full analysis set; HNSCC: Head and neck squamous cell carcinoma; PS: Performance status.

### Efficacy

At the data cutoff date of April 2, 2020, the median duration of SCT200 treatment was 14.2 weeks (95% CI: 6.3–18.4), with 44.4% (16/36) of patients receiving treatment for >18 weeks, while 25.0% (9/36) received treatment for 6–18 weeks. Seventy-five percent (27/36) of patients continued treatment without any dose interruptions, and 19.4% (7/36) experienced a single dose interruption.

**Figure 2:**
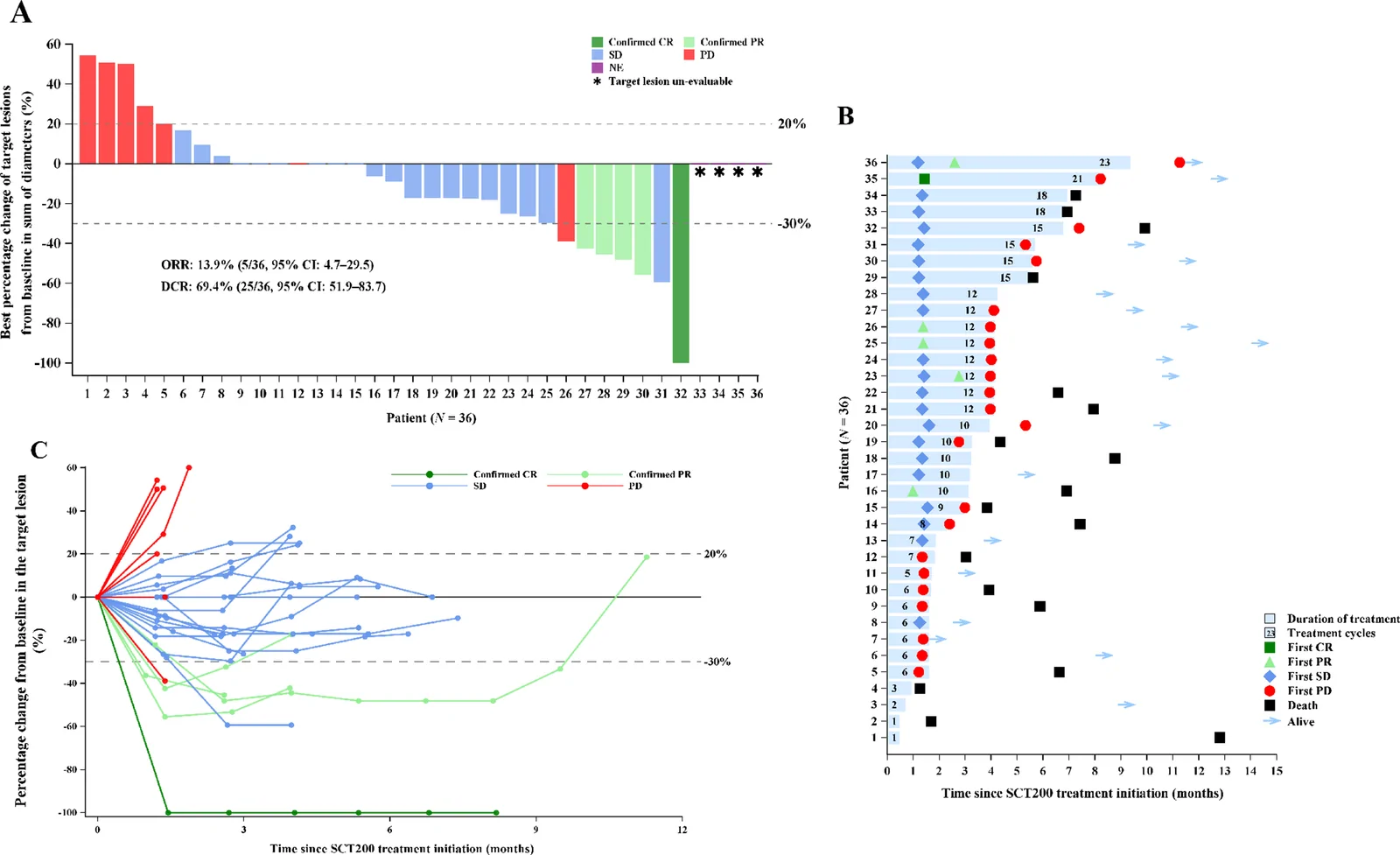
Individual patient data for efficacy and follow-up from the phase 2 trial of SCT200 in patients with platinum-refractory recurrent or metastatic HNSCC in the FAS. (A) Best percentage change from baseline in the sum of diameters of target lesions in each patient. (B) Swimming plot for all patients over time on study and overall treatment status. (C) Changes in target lesions from baseline in tumor size for all evaluable patients. CI: Confidence interval; CR: Complete response; DCR: Disease control rate; FAS: Full analysis set; HNSCC: Head and neck squamous cell carcinoma; NE: Not evaluable; ORR: Objective response rate; PD: Progressive disease; PR: Partial response; PS: Performance status; SD: Stable disease.

**Figure 3:**
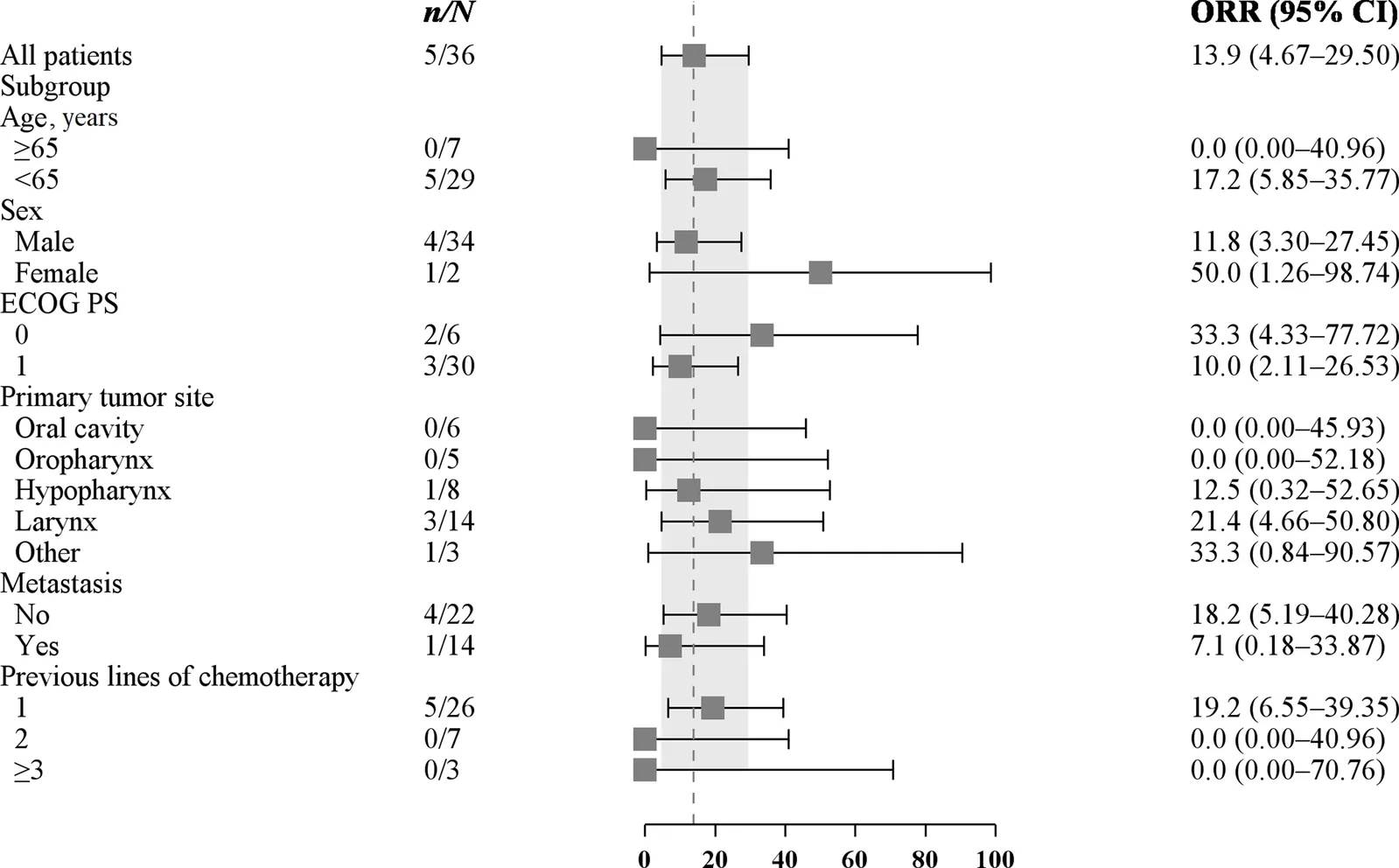
Forest plot of subgroup analysis in patients with platinum-refractory recurrent or metastatic HNSCC treated with SCT200 in the phase 2 trial in the FAS. CI: Confidence interval; ECOG: Eastern Cooperative Oncology Group; FAS: Full analysis set; HNSCC: Head and neck squamous cell carcinoma; ORR: Objective response rate; PS: Performance status.

The ORR was 13.9% (5/36, 95% CI: 4.7–29.5), with a CR rate of 2.8% (1/36), and a PR rate of 11.1% (4/36). Four patients were not evaluable and were considered to have no tumor response or SD, including one patient who refused imaging examination after SCT200 administration, one who discontinued SCT200 treatment after two doses due to acneiform dermatitis without a subsequent efficacy assessment, and two who died prior to the first efficacy assessment. The best overall response of SD was observed in 55.6% (20/36) of patients. Among these, 25.0% (5/20) maintained SD for more than 6 months, while 20.0% (4/20) were censored due to lack of subsequent efficacy assessment. The DCR was 69.4% (25/36, 95% CI: 51.9–83.7) [Figure 2A–C]. PRs were observed in patients with laryngeal and hypopharyngeal cancers. The ORR was 21.4% (3/14, 95% CI: 4.7–50.8) among patients with laryngeal cancer and 12.5% (1/8, 95% CI: 0.3–52.7) among those with hypopharyngeal cancer. Tumor response in other subgroups is presented in Figure 3. Among five patients who had achieved CR or PR, the median TTR was 1.38 months (95% CI: 0.99–2.60).

**Figure 4:**
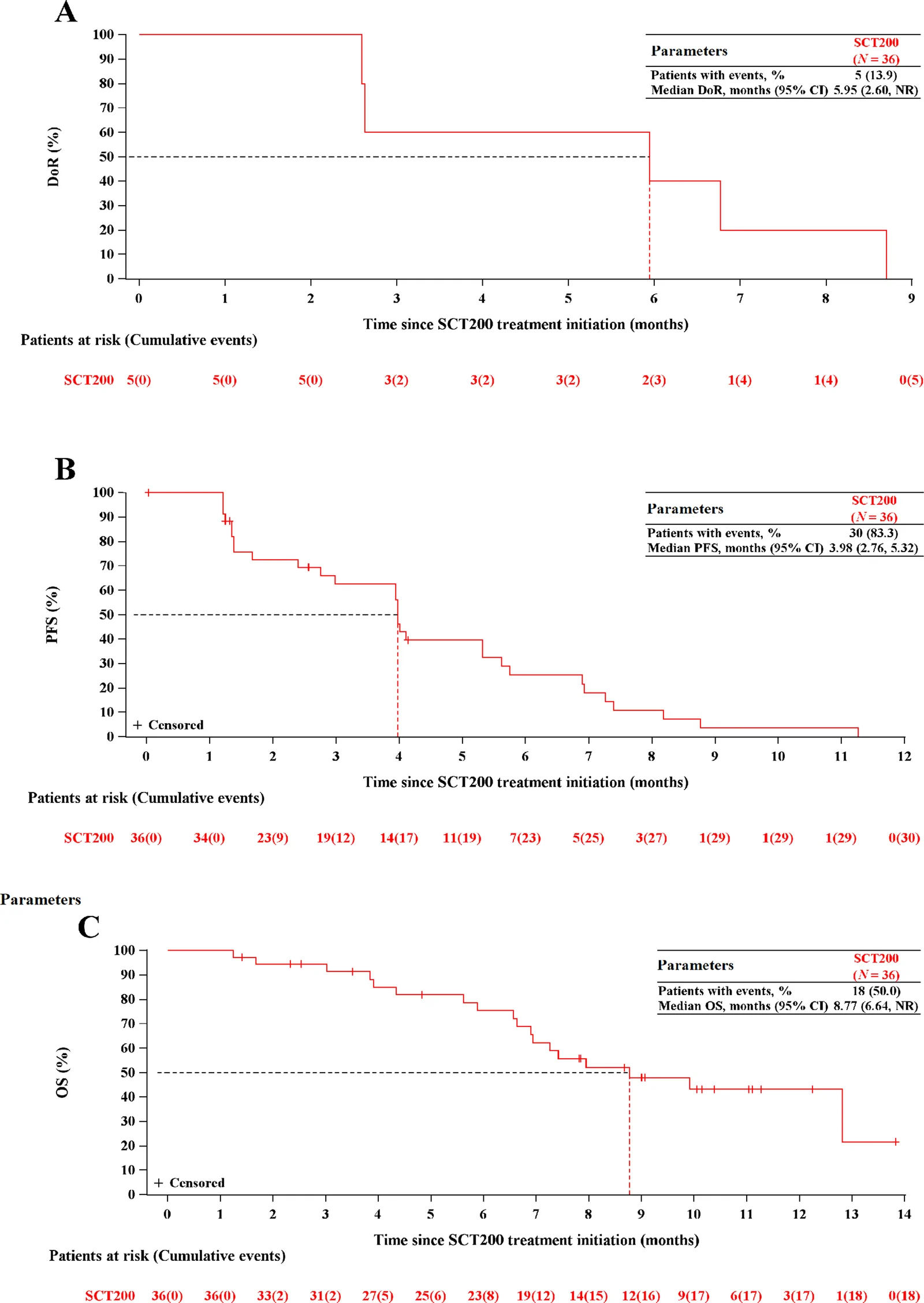
Kaplan–Meier curves for (A) DoR, (B) PFS, and (C) OS among patients with platinum-resistant recurrent or metastatic HNSCC treated with SCT200 in the full analysis set. CI: Confidence interval; DoR: Duration of response; HNSCC: Head and neck squamous cell carcinoma; PFS: Progression-free survival; OS: Overall survival; NR: Not reached.

After a median follow-up duration of 10.15 months (95% CI: 8.67–11.11), there were 30 PFS events and 18 deaths. The median DoR was 5.95 months (95% CI: 2.60–not reached [NR]) [Figure 4A]. The 6-month DoR rate was 40.0% (95% CI: 5.2–75.3). The median PFS was 3.98 months (95% CI: 2.76–5.32) [Figure 4B]. The 6-month PFS rate was 25.2% (95% CI: 11.5–41.6). Median PFS was 7.26 months (95% CI: 3.98–7.39) for oral cavity cancer, 3.98 months (95% CI: 2.40–NR) for oropharyngeal cancer, 1.68 months (95% CI: 1.22–6.90) for hypopharyngeal cancer, and 4.11 months (95% CI: 2.76–5.75) for laryngeal cancer. The median OS was 8.77 months (95% CI: 6.64–NR) [Figure 4C]. The 6-month OS rate was 75.3% (95% CI: 56.5–86.9), and the 12-month OS rate was 43.2% (95% CI: 24.7–60.4). Median OS was 7.95 months (95% CI: 1.25–12.81) for oral cavity, NR (95% CI: 6.57–NR) for oropharyngeal cancer, 8.77 months (95% CI: 1.68–NR) for hypopharyngeal cancer, and NR (95% CI: 5.62–NR) for laryngeal cancer.

### Safety

All patients (36/36) experienced treatment-emergent adverse events (TEAEs), and 94.4% (34/36) had treatment-related adverse events (TRAEs). The most common TRAEs included rash (20/36, 55.6%, 95% CI: 38.1–72.1), hypomagnesemia (19/36, 52.8%, 95% CI: 35.5–69.6), acneiform dermatitis (7/36, 19.4%, 95% CI: 8.2–36.0), hypokalemia (4/36, 11.1%, 95% CI: 3.1–26.1), hypophosphatemia (4/36, 11.1%, 95% CI: 3.1–26.1), and pyrexia (4/36, 11.1%, 95% CI: 3.1–26.1). 44.4% (16/36) of patients had ≥ grade 3 TRAEs, including hypomagnesemia (7/36, 19.4%, 95% CI: 8.2–36.0), hypokalemia (3/36, 8.3%, 95% CI: 1.8–22.5), rash (2/36, 5.6%, 95% CI: 0.7–18.7), and anemia (2/36, 5.6%, 95% CI: 0.7–18.7) [Table 2]. Among the five patients who had fatal AEs, one patient died of an unknown cause, for which the investigator was unable to determine its association with SCT200 and classified it as a TRAE. The remaining four deaths, attributed to dyspnea, cervical hemorrhage, or unspecified causes, were assessed by investigators as unrelated to SCT200. 22.2% (8/36) of patients had SCT200 treatment interruption, primarily due to hypomagnesemia (3/36, 8.3%) and hypokalemia (2/36, 5.6%). 16.7% (6/36) of patients had SCT200 treatment discontinuation, the reasons included infected neoplasm (1/36, 2.8%), tumor ulceration (1/36, 2.8%), hypomagnesemia (1/36, 2.8%), cholestatic jaundice (1/36, 2.8%), blood creatine phosphokinase MB increased (1/36, 2.8%), extradural hematoma (1/36, 2.8%), and hemorrhage (1/36, 2.8%), with hypomagnesemia assessed as possibly related to SCT200. 5.6% (2/36) of patients had SCT200 dose adjustments, and 5.6% (2/36) of patients had SCT200 infusion rate modifications.

**Table 2:** TRAEs of SCT200 in patients with platinum-refractory recurrent or metastatic HNSCC in the SS (*N* = 36).

TRAEs	All grades^a^		Grade ≥3	
	*n* (%)	95% CI	*n* (%)	95% CI
Rash	20 (55.6)	38.1–72.1	2 (5.6)	0.7–18.7
Hypomagnesemia	19 (52.8)	35.5–69.6	7 (19.4)	8.2–36.0
Acneiform dermatitis	7 (19.4)	8.2–36.0	1 (2.8)	0.1–14.5
Hypokalemia	4 (11.1)	3.1–26.1	3 (8.3)	1.8–22.5
Hypophosphatemia	4 (11.1)	3.1–26.1	0 (0.0)	–
Pyrexia	4 (11.1)	3.1–26.1	1 (2.8)	0.1–14.5
ALT increased	3 (8.3)	1.8–22.5	0 (0.0)	–
AST increased	3 (8.3)	1.8–22.5	0 (0.0)	–
Hepatic function abnormal	3 (8.3)	1.8–22.5	0 (0.0)	–
Paronychia	3 (8.3)	1.8–22.5	0 (0.0)	–
Dry skin	2 (5.6)	0.7–18.7	0 (0.0)	–
Hypocalcemia	2 (5.6)	0.7–18.7	1 (2.8)	0.1–14.5
Decreased appetite	2 (5.6)	0.7–18.7	0 (0.0)	–
Weight decreased	2 (5.6)	0.7–18.7	1 (2.8)	0.1–14.5
Blood bilirubin increased	2 (5.6)	0.7–18.7	1 (2.8)	0.1–14.5
Nausea	2 (5.6)	0.7–18.7	0 (0.0)	–
Hematuria	2 (5.6)	0.7–18.7	0 (0.0)	–
Anemia	2 (5.6)	0.7–18.7	2 (5.6)	0.7–18.7
Neck pain	2 (5.6)	0.7–18.7	0 (0.0)	–
Hypersensitivity	2 (5.6)	0.7–18.7	0 (0.0)	–

Note: The table lists all TRAEs of SCT200 that occurred in ≥2 patients within SS of individuals with platinum-refractory recurrent or metastatic HNSCC.

ALT: Alanine aminotransferase; AST: Aspartate aminotransferase; CI: Confidence interval; HNSCC: Head and neck squamous cell carcinoma; TRAEs: Treatment-related adverse events; SS: safety set; -: No data.

a Among all patients, one fatal TRAE was reported, in which the patient died of an unknown cause; the investigator could not determine its association with SCT200 and classified it as a TRAE.

Although no patient had a ≥20% decline in estimated glomerular filtration rate (eGFR) compared with baseline, serum magnesium levels declined progressively from 0.84 mmol/L at baseline to 0.53 mmol/L by week 25 [Table 3]. The absence of a correlation between magnesium loss and eGFR (*r* = −0.133, *p* = 0.126) indicates that hypomagnesemia was not driven by impaired glomerular filtration. These findings are consistent with inhibition of renal tubular magnesium reabsorption, a known on-target AE of anti-EGFR mAb therapy.^19^ Routine monitoring of magnesium and early supplementation are needed in the absence of renal impairment.

**Table 3:** Changes in serum creatinine, eGFR, and magnesium levels over time in patients with platinum-refractory recurrent or metastatic HNSCC treated with SCT200 in the phase 2 trial.

Parameters	Serum creatinine (μmol/L)	eGFR (mL·min-1·1.73 m^-2^)	Magnesium (mmol/L)
Baseline			
*n*	36	36	36
Mean (standard deviation)	74.1 (19.37)	93.5 (17.58)	0.84 (0.09)
Week 7			
*n*	25	25	25
Mean (standard deviation)	63.7 (13.28)	103.15 (11.78)	0.72 (0.147)
Change from baseline, mean (standard deviation)	−8.2 (9.48)	6.7 (8.71)	−0.12 (0.11)
Shapiro–Wilk test *p-value*	0.939	0.020	0.844
Student's *t*-test *p*-value	<0.001	<0.001	<0.001
Week 13			
*n*	21	21	21
Mean (standard deviation)	64.9 (13.19)	102.8 (15.29)	0.62 (0.172)
Change from baseline, mean (standard deviation)	−8.8 (13.99)	8.12 (12.83)	−0.23 (0.16)
Shapiro–Wilk *t*est *p*-value	0.610	0.409	0.626
Student's *t*-test *p*-value	0.009	0.009	<0.001
Week 19			
*n*	8	8	8
Mean (standard deviation)	68.5 (12.12)	96.13 (10.80)	0.59 (0.177)
Change from baseline, mean (standard deviation)	−8.9 (11.94)	8.11 (12.80)	−0.24 (0.13)
Shapiro–Wilk test *p-*value	0.234	0.553	0.722
Student's *t*-test *p*-value	0.074	0.061	0.002
Week 25			
*n*	6	6	6
Mean (standard deviation)	67.5 (15.21)	95.03 (14.35)	0.53 (0.19)
Change from baseline, mean (standard deviation)	−6.2 (10.32)	5.03 (7.40)	−0.29 (0.16)
Shapiro–Wilk test *p-*value	0.106	0.109	0.388
Student's *t*-test *p*-value	0.203	0.157	0.118

The eGFR was calculated using the 2021 CKD-EPI Adult eGFR Calculator. The normality test *p*-value for the eGFR Week 7 (*n* = 25) data was 0.0196 (<0.05), while the data from all other visit time points and baseline conformed to a normal distribution. Similarly, the data for Serum Creatinine and Magnesium at all visit time points and baseline also conformed to a normal distribution. Considering that the *t*-test is robust against moderate deviations from normality, especially when the sample size is relatively large, and to maintain consistency in statistical methods across all time points for ease of result comparison, we applied the *t*-test for all time points. eGFR: Estimated glomerular filtration rate; HNSCC: Head and neck squamous cell carcinoma.

The elevated incidence of rash and hypomagnesemia in this study is consistent with the known AE profile of other EGFR mAbs, and specific management guidelines for these AEs were provided within the study protocol. For ≥ grade 3 skin AEs: After the second occurrence, upon recovery to ≤ grade 2, the dose of SCT200 should be reduced to 80%; after the third occurrence, upon recovery to ≤ grade 2, the dose of SCT200 should be reduced to 60%; after the fourth occurrence, SCT200 treatment should be permanently discontinued. Additionally, if SCT200 dosing is delayed for more than two cycles and AE has not recovered to ≤ grade 2, SCT200 treatment should be permanently discontinued. For grade 2 hypomagnesemia, intravenous magnesium supplementation may be considered: for example, a 4.0 g once weekly injection of magnesium sulfate. In case of ≥ grade 3 hypomagnesemia, intravenous magnesium supplementation should be administered based on the severity of clinical symptoms: for example, a once-daily to twice-weekly injection of 4.0–10.0 g magnesium sulfate. If ≥ grade 3 hypomagnesemia persists despite aggressive magnesium replacement, SCT200 treatment should be discontinued.

## Discussion

Studies have consistently confirmed the efficacy of cetuximab in the treatment of patients with HNSCC.^5^, ^6^, ^7^ Cetuximab has been approved by the U.S. FDA for use in combination with radiation therapy for patients with locally or regionally advanced HNSCC, where it enhances the effects of radiotherapy by inhibiting EGFR-mediated tumor cell proliferation and survival.^20^ In addition, cetuximab is approved for use in first-line treatment of R/M HNSCC when combined with platinum-based chemotherapy, providing improved response rates and OS compared with chemotherapy alone.^5^ Moreover, cetuximab is indicated for the treatment of patients with R/M HNSCC whose disease has progressed following platinum-based chemotherapy, offering a targeted option for those who may not be eligible for or have failed other treatments.^21^ These approvals underscore the clinical importance of EGFR mAbs in treating HNSCC and highlight the ongoing need to explore and develop novel EGFR mAbs.

This study evaluated SCT200, a novel fully human anti-EGFR mAb, as a potential second-line treatment for Chinese patients with R/M HNSCC progressing after platinum-based therapy. Notably, the National Medical Products Administration (NMPA) has not approved cetuximab or other EGFR-targeting agents for second-line therapy in this patient population. As such, this study addresses a significant unmet clinical need by evaluating the potential of SCT200 to provide an effective treatment option for patients with limited alternatives when the disease progresses after platinum-based chemotherapy or is intolerable to chemotherapy.

In this study, the ORR was 13.9% (95% CI: 4.7–29.5), the DCR was 69.4% (95% CI: 51.9–83.7), the median PFS was 3.98 months (95% CI: 2.76–5.32), and the median OS was 8.77 months (95% CI: 6.64–NR). The approval of cetuximab as a second-line treatment for R/M HNSCC progressing after platinum-based therapy was based on an open-label, non-comparative, multicenter phase 2 study. This study enrolled patients with R/M HNSCC who had documented disease progression following platinum-based therapy.^21^ In that study, patients received cetuximab intravenously, starting with a dose of 400 mg/m^2^, followed by weekly infusions of 250 mg/m^2^. Among 103 evaluable patients, the ORR was 13% (95% CI: 7–21), the DCR was 46% (95% CI: 36–56), the median time to progression was 70 days, and the median survival time was 178 days.^21^

Among international studies—including CheckMate-141, LUX-Head & Neck 1, BERIL-1, and the PRISM studies—the reported distributions of primary tumor sites were as follows: 14%–25% for laryngeal cancer, 2–19% for hypopharyngeal cancer, 28%–49% for oral cavity cancer, and 28–35% for oropharyngeal cancer, 22, 23, 24, 25 39%, 22%, 17%, and 14% for respective cancer types in our study. Our study enrolled a higher proportion of patients with laryngeal and hypopharyngeal cancers, and a lower proportion of those with oral cavity and oropharyngeal cancers, compared to international studies. SCT200 showed promising efficacy in laryngeal cancer, which is relatively common in China, with an ORR of 21.40% (3/14; 95% CI: 4.7–50.8), a median PFS of 4.11 months (95% CI: 2.76–5.75), and a median OS that was NR (95% CI: 5.62–NR). Anti-tumor activity was also observed in hypopharyngeal cancer, with an ORR of 12.5% (1/8; 95% CI: 0.3–52.7), a median PFS of 1.68 months (95% CI: 1.22–6.90), and a median OS of 8.77 months (95% CI: 1.68–NR). In contrast, no objective responses were observed in the small subgroups of patients with oral cavity (0/6) or oropharyngeal cancer (0/5). The lack of observed efficacy in these cancer types is likely attributable to their limited sample sizes.

SCT200 showed numerically improved efficacy outcomes compared with historical single-agent chemotherapy, the standard of care for R/M HNSCC progressing after platinum-based therapy. In the BERIL-1 study, treatment with paclitaxel monotherapy resulted in a median PFS of 3.5 months (95% CI: 2.2–3.7) and a median OS of 6.5 months (95% CI: 5.3–8.8) in patients with platinum-pretreated R/M HNSCC.^23^ Similarly, in patients with platinum-refractory R/M HNSCC, docetaxel monotherapy was associated with a median PFS of 2.1 months (95% CI: 1.8–2.4) and a median OS of 6.7 months (95% CI: 2.5–10.8).^26^ In the LUX-Head and Neck 3 study, methotrexate monotherapy yielded a median PFS of 2.6 months (95% CI: 1.5–2.8) and a median OS of 6.4 months (95% CI: 5.2–8.2) in patients with R/M HNSCC progressing upon or after platinum-based chemotherapy.^27^

The PD-1 mAbs nivolumab and pembrolizumab have been approved as monotherapies for second-line treatment of R/M HNSCC.^24^^,^^28^ In CheckMate 141 and KEYNOTE-040 studies, nivolumab and pembrolizumab demonstrated ORRs of 13.3% (32/240, 95% CI: 9.3–18.3) and 14.6% (36/247, 95% CI: 10.4–19.6), respectively. The median PFS was 2.0 months (95% CI: 1.9–2.1) for nivolumab and 2.1 months (95% CI: 2.1–2.3) for pembrolizumab. The median OS was 7.5 months (95% CI: 5.5–9.1) for nivolumab and 8.4 months (95% CI: 6.4–9.4) for pembrolizumab. However, among patients with low PD-L1 expression (TPS≤1% or CPS<1), efficacy was notably reduced, with ORRs of 12.3% (9/73) for nivolumab and 4.0% (2/50, 95% CI: 0.5–13.7) for pembrolizumab, a median OS of 5.7 months (95% CI: 4.4–12.7) for nivolumab and 6.3 months (95% CI: 3.8–8.9) for pembrolizumab. These findings emphasize the reliance of PD-1 mAb efficacy on PD-L1 expression. Compared with these agents, SCT200 demonstrated a numerically longer median PFS with comparable ORR and OS in an unselected patient population compared with these agents. PD-L1-negative patients account for 20%–30% of patients with R/M HNSCC, and SCT200 provides a potential new treatment option for this patient population.^24^^,^^28^ Additionally, PD-1 mAbs exhibit a delayed onset of response, reflected in median TTR of 2.1 months for nivolumab and 4.5 months (Interquartile range 2.3–6.4) for pembrolizumab.^24^^,^^28^ SCT200 achieved a median TTR of 1.38 months (95% CI: 0.99–2.60), suggesting a more rapid clinical benefit. In terms of safety, PD-1 mAbs are commonly associated with immune-related AEs, including hypothyroidism, gastrointestinal toxicities, and pneumonitis.^24^^,^^28^ By contrast, SCT200 was mainly associated with skin AEs such as rash and acneiform dermatitis, as well as electrolyte disturbances, including hypomagnesemia and hypokalemia. These differences suggest that SCT200 may offer an alternative treatment option, particularly for patients with low or negative PD-L1 expression.

With the approval of immunotherapies and the development of more innovative agents, the use of single-agent therapy in clinical practice for platinum-resistant R/M HNSCC has likely declined. Data from recent studies illustrate the current therapeutic landscape. In a retrospective Chinese study of 38 patients with platinum-resistant R/M HNSCC who received paclitaxel and ifosfamide combined with immunotherapy and/or cetuximab, the reported outcomes included an ORR of 15.8% (6/38, 95% CI: 4.2–27.4), a DCR of 36.8% (14/38, 95% CI: 21.5–52.1), a median PFS of 3.3 months (95% CI: 2.99–3.7), and a median OS of 9.6 months (95% CI: 3.5–15.7).^29^ Similarly, in a phase II study of 106 patients with R/M HNSCC (including 34% Asian patients), the ORR was 9.9% (7/71, 95% CI: 4.1–19.3) for cetuximab combined with PI3K inhibitor alpelisib and 5.7% (2/35, 95% CI: 0.7–19.2) for cetuximab monotherapy, the DCR was 43.7% (31/71, 95% CI: 31.9–56.0) and 28.6% (10/35, 95% CI: 14.6–46.3), the median PFS was 86.0 days (95% CI: 73.0–135.0) and 87.0 days (95% CI: 49.0–133.0) (HR 1.12, 95% CI: 0.69–1.82), and the median OS was 173.0 days (95% CI: 142.0–249.0) and 263.0 days (95% CI:181.0–444.0) (HR 1.28, 95% CI: 0.80–2.05), respectively.^30^ Another phase II study of the nanoliposomal irinotecan plus 5-fluorouracil and leucovorin regimen in 43 patients, demonstrating an ORR of 11.6% (5/43), a DCR of 65.1% (28/43), a median PFS of 2.7 months (95% CI: 1.5–3.2), and a median OS of 8.1 months (95% CI: 5.1–10.5).^31^ Furthermore, in the TACTI-002 Part C study, the combination of eftilagimod alpha (a soluble LAG-3 protein) and pembrolizumab yielded an ORR of 24% (9/37), a median PFS of 2.1 months, and a median OS of 8.7 months in evaluable patients with metastatic HNSCC progressing after first-line platinum-based therapy.^32^ Compared with these regimens, SCT200 monotherapy in our study demonstrated comparable efficacy, underscoring its potential clinical value in this difficult-to-treat patient population. Notably, another innovative agent, petosemtamab—a novel, first-in-class EGFR × LGR5 bispecific antibody—has shown more promising activity. In a phase II study evaluating petosemtamab monotherapy in patients who had received at least one prior systemic therapy, among 75 evaluable patients with R/M HNSCC, the ORR was 36% (27/75, 95% CI: 27–46), with a median PFS of 4.9 months (95% CI: 3.2–5.4) and a median OS of 11.4 months (95% CI: 7.2–15.9).^33^ The promising efficacy of petosemtamab further validates the therapeutic potential of targeting EGFR in HNSCC.

While EGFR mAb monotherapy represents a valid treatment option for R/M HNSCC progressing after platinum-based therapy, its limitations include the potential development of therapeutic resistance and outcomes that are often suboptimal compared with those of combination therapies. Previous studies have established that the IgG1 isotype mAb cetuximab is among the most potent inducer of ADCC. Specifically, the crystallizable fragment region of cetuximab activates natural killer cells, leading to the lysis of tumor cells and the release of tumor-associated antigens. These antigens are then captured and presented by dendritic cells and macrophages, which in turn prime cytotoxic T cells and engage the immune system. This mechanism provides a rationale for combining cetuximab with PD-1/PD-L1 mAbs to achieve synergistic anti-tumor activity.^34^ Supporting this approach, A phase 1b/2 study evaluating PD-1 mAb toripalimab in combination with cetuximab demonstrated promising outcomes in patients with platinum-refractory R/M HNSCC, with an ORR of 60% (95% CI 44.3–74.3), a median PFS of 9.9 months (95% CI 4.2–NA), and a median OS of 15.4 months (95% CI 8.5–17.7).^35^ Future studies will explore the clinical efficacy of combining SCT200 with PD-1 inhibitors in R/M HNSCC. Additionally, the combination of PD-1 inhibitors and anti-angiogenic therapy, such as penpulimab plus anlotinib, has also demonstrated promising results in patients with R/M HNSCC after failure of platinum-based chemotherapy.^36^ Collectively, these findings suggest that combination strategies may provide more robust and durable efficacy than monotherapy for this patient population.

This study has several limitations. First, this was a single-arm phase 2 study conducted without a concurrent control group; therefore, the observed efficacy could have been influenced by factors such as patient heterogeneity or the natural disease kinetics following platinum therapy, rather than being attributable solely to SCT200 treatment. Second, the sample size was small, which limits the statistical power of the analyses. Third, no exploratory biomarker analyses were conducted.

In conclusion, this is the first prospective study of EGFR mAb monotherapy demonstrating promising efficacy and a manageable safety profile of SCT200 in Chinese patients with R/M HNSCC progressing after platinum-based chemotherapy. This study addresses a critical evidence gap and positions SCT200 as a potential treatment option in the second-line setting. These findings warrant further investigation in larger, randomized controlled trials to confirm the efficacy and safety of SCT200 and to identify predictive biomarkers in this patient population.

## Authors contribution

Yuankai Shi was the principal investigator, contributing to study design, supervision, coordination, patient enrollment, patient management, manuscript writing, revisions, and editing; Qingyuan Zhang, Wei Wang, Jianhua Shi, Xiaodong Zhu, Kunyu Yang, Lin Wang, Guoqing Li, Peiguo Wang, and Lin Gui were investigators in this study, and contributed to patient enrollment and patient management. All authors reviewed and approved the final version of the manuscript and agreed to submit it for publication.

## Ethics statement

This study was conducted in accordance with the ethical principles of the *Declaration of Helsinki* and the International Council for Harmonization of Technical Requirements for Pharmaceuticals for Human Use (ICH) Guideline for Good Clinical Practice. The clinical study protocol was reviewed and approved by the Institutional Review Board or Ethics Committee of the National Cancer Center/Cancer Hospital, Chinese Academy of Medical Sciences, and Peking Union Medical College (Approval No. 18–125/1703), and by the Ethics Committees of all participating hospitals. Prior to any study-related procedures, written informed consent was obtained from all individual participating patients included in this study.

## Data availability statement

The authors declare that the data supporting the findings of this study are available within the main manuscript. Correspondence and requests for materials should be addressed to the corresponding author (Professor Yuankai Shi).

## Declaration of generative AI and AI-assisted technologies in the writing process

During the preparation of this work, the author(s) did not use any generative AI or AI-assisted technologies for writing, content generation, data analysis, or interpretation. The entire process of drafting, writing, and revising the manuscript was solely performed by the human author(s), who take full responsibility for the content and integrity of this work.

## Funding

This study was sponsored by Sinocelltech Ltd, which provided the investigational drug SCT200 and was involved in the study design in collaboration with the academic investigators. The study was also partly supported by the National Science and Technology Major Projects of China (No. 2019ZX09732-001).

## Conflict of interest

The authors declare that they have no known competing financial interests or personal relationships that could have appeared to influence the work reported in this paper.

## References

[1] Bray F., Laversanne M., Sung H. (2024). Global cancer statistics 2022: GLOBOCAN estimates of incidence and mortality worldwide for 36 cancers in 185 countries. CA Cancer J Clin.

[2] Xia C., Dong X., Li H. (2022). Cancer statistics in China and United States, 2022: profiles, trends, and determinants. Chin Med J.

[3] Chow L.Q.M. (2020). Head and neck cancer. N Engl J Med.

[4] Argiris A., Karamouzis M.V., Raben D., Ferris R.L. (2008). Head and neck cancer. Lancet.

[5] Vermorken J.B., Mesia R., Rivera F. (2008). Platinum-based chemotherapy plus cetuximab in head and neck cancer. N Engl J Med.

[6] Guo Y., Shi M., Yang A. (2015). Platinum-based chemotherapy plus cetuximab first-line for Asian patients with recurrent and/or metastatic squamous cell carcinoma of the head and neck: results of an open-label, single-arm, multicenter trial. Head Neck.

[7] Guo Y., Luo Y., Zhang Q. (2021). First-line treatment with chemotherapy plus cetuximab in Chinese patients with recurrent and/or metastatic squamous cell carcinoma of the head and neck: efficacy and safety results of the randomised, phase III CHANGE-2 trial. Eur J Cancer.

[8] Burtness B., Harrington K.J., Greil R. (2019). Pembrolizumab alone or with chemotherapy versus cetuximab with chemotherapy for recurrent or metastatic squamous cell carcinoma of the head and neck (KEYNOTE-048): a randomised, open-label, phase 3 study. Lancet.

[9] Shi Y., Guo W., Wang W. (2024). Finotonlimab with chemotherapy in recurrent or metastatic head and neck cancer: a randomized phase 3 trial. Nat Med.

[10] National Comprehensive Cancer Network (NCCN) (2025).

[11] Yarden Y., Shilo B.Z. (2007). SnapShot: EGFR signaling pathway. Cell.

[12] Cancer Genome Atlas Network (2015). Comprehensive genomic characterization of head and neck squamous cell carcinomas. Nature.

[13] Johnson D.E., Burtness B., Leemans C.R., Lui V.W.Y., Bauman J.E., Grandis J.R. (2020). Head and neck squamous cell carcinoma. Nat Rev Dis Primers.

[14] Kalyankrishna S., Grandis J.R. (2006). Epidermal growth factor receptor biology in head and neck cancer. J Clin Oncol.

[15] Bai M., Wang M., Deng T. (2022). Safety and efficacy of anti-EGFR monoclonal antibody (SCT200) as second-line therapy in advanced esophageal squamous cell carcinoma. Cancer Biol Med.

[16] Zhang W., Han X., Yang L. (2022). Safety, pharmacokinetics and efficacy of SCT200, an anti-EGFR monoclonal antibody in patients with wild-type KRAS/NRAS/BRAF metastatic colorectal cancer: a phase I dose-escalation and dose-expansion study. BMC Cancer.

[17] Yang L., Zhang W., Fan N. (2024). Efficacy, safety and genomic analysis of SCT200, an anti-EGFR monoclonal antibody, in patients with fluorouracil, irinotecan and oxaliplatin refractory RAS and BRAF wild-type metastatic colorectal cancer: a phase Ⅱ study. EBioMedicine.

[18] Lala M., Chirovsky D., Cheng J.D., Mayawala K. (2018). Clinical outcomes with therapies for previously treated recurrent/metastatic head-and-neck squamous cell carcinoma (R/M HNSCC): a systematic literature review. Oral Oncol.

[19] Ortega B., Dey J.M., Gardella A.R., Proano J., Vaneerde D. (2017). Antibody-mediated inhibition of EGFR reduces phosphate excretion and induces hyperphosphatemia and mild hypomagnesemia in mice. Phys Rep.

[20] Bonner J.A., Harari P.M., Giralt J. (2006). Radiotherapy plus cetuximab for squamous-cell carcinoma of the head and neck. N Engl J Med.

[21] Vermorken J.B., Trigo J., Hitt R. (2007). Open-label, uncontrolled, multicenter phase II study to evaluate the efficacy and toxicity of cetuximab as a single agent in patients with recurrent and/or metastatic squamous cell carcinoma of the head and neck who failed to respond to platinum-based therapy. J Clin Oncol.

[22] Rischin D., Spigel D.R., Adkins D. (2016). PRISM: phase 2 trial with panitumumab monotherapy as second-line treatment in patients with recurrent or metastatic squamous cell carcinoma of the head and neck. Head Neck.

[23] Soulières D., Faivre S., Mesía R. (2017). Buparlisib and paclitaxel in patients with platinum-pretreated recurrent or metastatic squamous cell carcinoma of the head and neck (BERIL-1): a randomised, double-blind, placebo-controlled phase 2 trial. Lancet Oncol.

[24] Ferris R.L., Blumenschein Jr G., Fayette J. (2016). Nivolumab for recurrent squamous-cell carcinoma of the head and neck. N Engl J Med.

[25] Machiels J.P.H., Haddad R.I., Fayette J. (2015). Afatinib versus methotrexate as second-line treatment in patients with recurrent or metastatic squamous-cell carcinoma of the head and neck progressing on or after platinum-based therapy (LUX-head & neck 1): an open-label, randomised phase 3 trial. Lancet Oncol.

[26] Cho B.C., Keum K.C., Shin S.J. (2009). Weekly docetaxel in patients with platinum-refractory metastatic or recurrent squamous cell carcinoma of the head and neck. Cancer Chemother Pharmacol.

[27] Guo Y., Ahn M.J., Chan A. (2019). Afatinib versus methotrexate as second-line treatment in Asian patients with recurrent or metastatic squamous cell carcinoma of the head and neck progressing on or after platinum-based therapy (LUX-head & neck 3): an open-label, randomised phase III trial. Ann Oncol.

[28] Cohen E.E., Soulières D., Le Tourneau C. (2019). Pembrolizumab versus methotrexate, docetaxel, or cetuximab for recurrent or metastatic head-and-neck squamous cell carcinoma (KEYNOTE-040): a randomised, open-label, phase 3 study. Lancet.

[29] Chou M.Y., Wu W.C., Chu P.Y. (2024). Paclitaxel-Ifosfamide-based therapy as salvage treatment in platinum-resistant recurrent/metastatic head and neck cancer. In Vivo (Athens).

[30] Abdul Razak A.R., Wang H.M., Chang J.Y. (2023). A phase 1b/2 study of alpelisib in combination with cetuximab in patients with recurrent or metastatic head and neck squamous cell carcinoma. Targeted Oncol.

[31] Yang M.H., Wu S.Y., Ho I.W. (2025). Nanoliposomal irinotecan in combination with 5-Fluorouracil and leucovorin for advanced head and neck and esophageal squamous cell carcinoma after prior platinum-based chemotherapy or chemoradiotherapy: a multicenter phase II trial. Cancer Med.

[32] Doger de Spéville B., Felip E., Forster M. (2023). Final results from TACTI-002 part C: a phase II study of eftilagimod alpha (soluble LAG-3 protein) and pembrolizumab in patients with metastatic 2nd line head and neck squamous cell carcinoma unselected for PD-L1. J Clin Oncol.

[33] Le Tourneau C., Fayette J., Even C. (December 7, 2024).

[34] Della Corte C.M., Fasano M., Ciaramella V. (2022). Anti-tumor activity of cetuximab plus avelumab in non-small cell lung cancer patients involves innate immunity activation: findings from the CAVE-lung trial. J Exp Clin Cancer Res.

[35] Guo Y., Li Z., Hu D. (2023). Updated safety and efficacy of toripalimab combined with cetuximab in platinum-refractory recurrent or metastatic head and neck squamous cell carcinoma (R/M-HNSCC): a phase Ib/II clinical trial. J Immunother Cancer.

[36] Shi Y., Gao L., Tian Y. (2023). Penpulimab combined with anlotinib in patients with R/M HNSCC after failure of platinum-based chemotherapy: a single-arm, multicenter, phase Ⅱ study. ESMO Open.

